# An Intelligent Cost-Efficient System to Prevent the Improper Posture Hazards in Offices Using Machine Learning Algorithms

**DOI:** 10.1155/2022/7957148

**Published:** 2022-08-18

**Authors:** Jehangir Arshad, Hafiza Mahnoor Asim, Muhammad Adil Ashraf, Mujtaba Hussain Jaffery, Khurram Shabih Zaidi, Melkamu Deressa Amentie

**Affiliations:** ^1^Department of Electrical and Computer Engineering, COMSATS University Islamabad, Lahore 54000, Pakistan; ^2^Department of Information Technology, Assosa University, Assosa 5220, Ethiopia

## Abstract

In this research, an intelligent and cost-efficient system has been proposed to detect the improper sitting posture of a person working at a desk, mostly in offices, using machine learning classification techniques. The current era demands to avoid the harms of an improper posture as it, when prolonged, is very painful and can be fatal sometimes. This study also includes a comparison of two arrangements. Arrangement 01 includes six force-sensitive resistor (FSR) sensors alone, and it is less expensive. Arrangement 02 consists of two FSR sensors and one ultrasonic sensor embedded in the back seat of a chair. The K-nearest neighbor (KNN), Naive Bayes, logistic regression, and random forest algorithms are used to augment the gain and enhanced accuracy for posture detection. The improper postures recognized in this study are backward-leaning, forward-leaning, left-leaning, and right-leaning. The presented results validate the proposed system as the accuracy of 99.8% is achieved using a smaller number of sensors that make the proposed prototype cost-efficient with improved accuracy and lower execution time. The proposed model is of a dire need for employees working in offices or even at the residential level to make it convenient to work for hours without having severe effects of improper posture and prolonged sitting.

## 1. Introduction and Literature Review

The economic growth of any country around the globe is directly linked to the number of employees working in offices as it increases GDP, however, working nonstop sitting on office chairs can cause actual harm to mental and physical health. According to a survey, 60% of Americans experience real health problems because of the use of technology during the day, including insomnia, eye strain, headache, and more, out of which 30 percent have severe back pain and 27 percent have neck pain because of prolonged sitting in an improper posture [1]. Moreover, if a person sits on a chair in the wrong posture for a long time, it can cause more severe and painful diseases, such as pressure ulcers, back pain, spinal dysfunction, joint degeneration, rounded shoulders, and a potbelly. The wrong posture causes an increase in pressure, friction, and shear on the chair, and rubbing the chair with the skin for a long time can tear the skin apart, resulting in severe pressure ulcers, leading to death.

In general, the office timings are eight hours, and people also work overtime or work longer than that without noticing their improper sitting patterns. For example, sitting eight hours on a chair with no physical activity can increase pressure at specific points in the body. The continuous rubbing of the skin on the chair surfaces results in pressure ulcers and other joint pains. Moreover, if the table height and distance from the table to a chair are not correct, the person has to bend toward his laptop, making a curve in his spine. These body curves can cause many hazards, such as back pain. Such improper postures, when taken for a prolonged period, can cause severe damage to health, and they must be prevented.

A good working environment is crucial in helping employees put their best foot forward. Sit-stand working stations provide a good environment and reduce discomfort. Studies conclude that sit-stand working stations reduce perceived discomfort and increase productivity [2]. Sitting on the chair in an awkward posture or in one posture for a long time will cause harmful diseases, including pressure ulcers. The severe impacts of improper posture increased the importance of pressure recognition systems. There are many methods and techniques to detect the wrong posture to prevent getting improper posture and severe effects. One of the highest accuracy techniques to detect posture is FSR sensors and machine learning. Scientists and researchers have done much research on detecting posture accurately, mainly for wheelchair users. In addition to the traditional algorithms of the Naive Bayes classifier (NB), decision tree (DT), neural network (NN), multinomial logistic regression (MLR), and support vector machine, a system was developed to classify the sitting postures using different machine learning algorithms, such as the convolutional neural network (CNN) algorithm (SVM). A sensing cushion was created by installing a pressure sensor mat (8 8) within the children's chair seat cushion [3]. A pressure mapping device was used to measure the pressure in the right, one-sided prone, chin supported, and slumped sitting positions [4]. The WDI (XAP) found a statistically significant difference between the three incorrect sitting postures (one side prone, chin propped, and slumped sitting) and the correct sitting posture (one side prone, chin propped, and slumped sitting) [5]. An embedded device was utilized to acquire position-related information using a machine learning method termed dimensionality reduction (DR) and classification. The system includes a DR step based on the principal component analysis (PCA) [6]. For pose detection between the matrix stored (in the system) and new data obtained by pressure and distance sensors, K-nearest neighbours (KNN) classifiers are used [7–13]. Noninvasive optical fiber sensor architecture can be fitted to the bottom of a shoe for remote plantar pressure monitoring, which might be utilized in an IoT e-Health system to track people's health. The study looks at creating an optical fiber sensor multiplexed network (using fiber bragg gratings) to measure the distribution of foot plantar pressure during locomotion (walking) [8].

Picture data from the posture was captured using a film-type pressure sensor. The study enlisted the participation of twenty-six children, who were photographed in seven distinct poses. The authors employed a seven-layer convolutional neural networks (CNN) technique. In addition, the artificial neural networks (ANN) approach, one of the machine learning techniques, was utilized to compare classification accuracy [14–20]. The system (FPGA) is made of six flex sensors, an ADC board, and a machine learning algorithm based on a two-layer artificial neural network (ANN) developed on a Spartan-6 field programmable gate array [10]. The system achieves 97.78 percent accuracy with a floating-point evaluation and 97.43 percent accuracy with a 9-bit fixed-point implementation. The maximum propagation delay for the ANN and ADC control logic is 8.714 ns [11]. Ahmad et al. employed the J48 algorithm to identify the five types of sitting postures using the pressure readings of twelve pressure sensors as features, achieving 99.47 percent experimental classification accuracy [12]. A center of pressure, contact area proportion, and pressure ratios are used in another article to identify five common trunk postures, two common left foot postures, and three common right foot postures. Lower-resolution mapping characteristics were compared to high-resolution sensor pressure mats on the backrest and seat pan features [21–26]. To recognize the postures of each body component, five distinct supervised machine-learning approaches are used [13]. Using a specific hardware system that interprets video in real-time using convolutional neural networks, a system based on the worker's postural detection is created, constructed, and tested. This device can identify the worker's neck, shoulders, and arms position and provide advice to help them avoid health problems caused by bad posture [12]. Many additional efforts, such as clinical implication assessment for diabetes mellitus [27], are built on machine learning techniques. There are also a slew of additional classification-based efforts to resolve health difficulties [28]. An intelligent way to forecast the accuracy of a model is to use an artificial neural network [29]. Multiple approaches are used in previous studies for the detection of posture, however, in this work, the cost is considered the main factor, and the higher accuracy of the system is achieved. Moreover, the proposed system is portable and multiple configurations of sensors are tested in this system [30].  Table 1 shows a comparison of prior research literature reviews. A system is designed to measure the variation in FSR sensor data on a chair according to the changing of the body sitting positions and the collection of that data.

- Two arrangements have been used in this study to find the cost-efficient and higher accuracy arrangement of sensors according to the weight distribution of a sitting person on a chair
- Different machine learning techniques are used to clean the dataset to make it more efficient and useful
- Four machine learning algorithms are applied to detect the posture of a sitting person

The paper is divided into the following sections: designed system, materials and methods, hardware, software, configurations and specifications, data processing, results and discussion, conclusion, and future work.

## 2. Designed System

**Figure 1:**
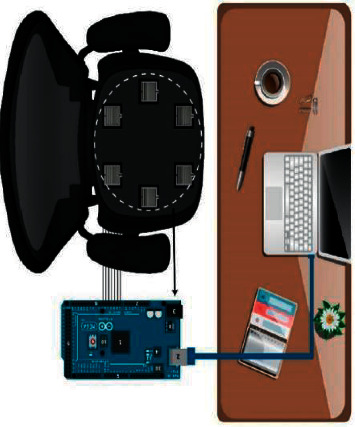
System diagram. FSR sensor data are being sent to the laptop through Arduino serial communication.

The sensors are connected to Arduino, and then the dataset is generated through serial communication, as shown in Figure 1. The data is stored in an excel file. In this way, data is stored, and then four different machine learning algorithms are applied to this data. This system supports mainly the table working in offices or at home. While working on laptops or computers in an office or at home, posture can easily be detected, and a warning of improper posture will be given on the laptop when that posture is taken for a prolonged period of time. The FSR sensors are very thin and small that they cannot be noticed, or no one can be annoyed or disturbed by these sensors. Hence, it is an easy and efficient system to detect a person's posture when working on a table. It is a regular practice that people bend over to focus on the screen, and it can impact their health badly and have severe health hazards. Thus, this system makes posture detection more straightforward and efficient.

## 3. Materials and Methods

**Figure 2:**
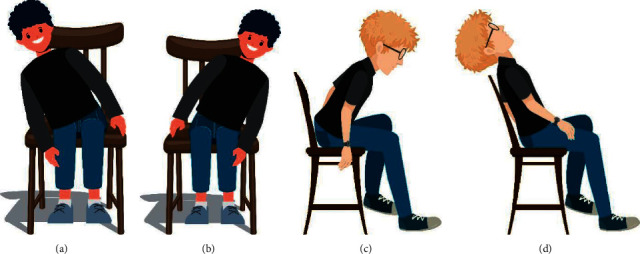
Four improper postures, namely left-leaning, right-leaning, forward-leaning, and backward leaning (from left to right).

The process of posture detection can be done in various ways and methods, one of which is the approach discussed in this paper, which is the analysis and computation of pressure distribution data on a chair. In this approach, the force resistive sensors FSR are placed in a meaningful manner on a standard ordinary office chair, and when a person sits on it, the pressure distribution on the chair is measured by the sensors, and the posture is predicted using machine learning algorithms. In this paper, four machine learning algorithms are applied, including logistic regression, KNN, random forest, and Naïve Bayes, to predict the four improper sitting patterns, such as bending forward, bending backward, bending left, and bending right, as in Figure 2.

### 3.1. System

This study uses FSR (force-sensitive resistor) sensors that exhibit varying degrees of resistance to force directly proportional to the pressure exerted on them. Firstly, the FSR sensors are used to measure the pressure difference according to weight distribution on a chair. Then, this data is refined into a functional form to apply machine learning algorithms for the prediction or detection of the posture. The embedded system consists of two main parts, such as hardware and software.

#### 3.1.1. Hardware

**Figure 3:**
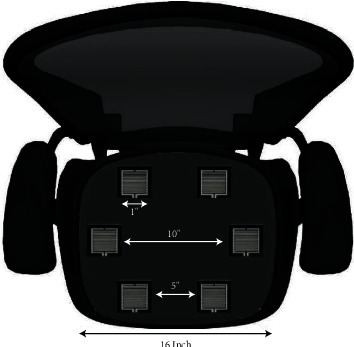
Arrangement 01, six sensors arrangement on a chair.

The weight distribution is evenly or unevenly divided on a chair when a person sits on it according to its sitting position. Hence, the pressure distribution on a chair is linked to a person's body mass and weight. Therefore, for the better capturing of pressure sensor data, the placement of FSR sensors must be meaningful. In this paper, two sensor placements are discussed to study the change in accuracy by changing the sensor arrangement. The two arrangements of sensors are as follows:

*(1) Arrangement 01*. In this arrangement, six FSR sensors are placed to cover the main pressure points of the human body, such as under the thighs, hip joints, and sides, as shown in Figure 3. When a person leans to the right, the right sensors have a higher-pressure value than the left ones and vice versa. In this way, the wrong posture is detected through the pressure distribution technique. The sensors work in a pair as the front two sensors are mainly used to detect the forward-leaning, and backward leaning is also detected by two sensors. When the pressure on the front two sensors increases, the person is sitting, leaning forward, and it is similar to backward leaning. Similarly, the left and right sensors are used to detect a person's left- and right-leaning positions. The chair used in this research is 16 × 16 inches in size, and the vertical distance between the forward and backward pair of sensors is 5 inches, while the left-right sensors are embedded at edges, with a distance of 10 inches. The FSR sensor used in this study is approximately 1 inch long.

*(2) Arrangement 02*. The need for a second arrangement arises because of the higher cost of FSR sensors. In this approach, an ultrasonic sensor is used instead of FSR sensors for backward or forward-leaning detection.

**Figure 4:**
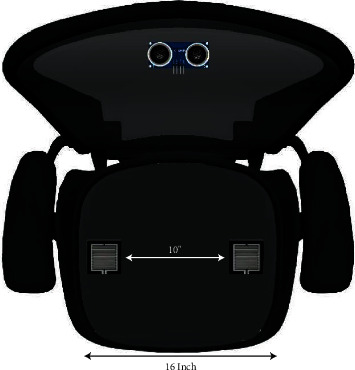
Arrangement 02: two FSR and one ultrasonic sensor.

The optimal range for ideal posture is set on an ultrasonic sensor, and when the range exceeds, the forward-leaning posture is detected, and when the distance between the ultrasonic sensor and the person reduces to zero, it is predicted as a backward leaning position. Arduino mega is used for arrangement 01 because of the more significant number of FSR sensors, and Arduino UNO is used for arrangement 02 because of the smaller number of FSR inputs. For this reason, arrangement 02 is considered cost-efficient. Moreover, FSR sensors are reduced to more than half in arrangement 02.  Figure 4 shows two FSR and one Ultrasonic sensor deployed in arrangement 2. A laptop processor is used for machine learning algorithms and Arduino serial communication.  Table 2 shows hardware and specifications used for the proposed system.

The cost comparison of arrangement 01 and arrangement 02 is given in Table 3.

#### 3.1.2. Software

The sensor data is sent to Arduino IDE via serial communication, and then Excel is used for storing the collected data. The Google collab notebook is used for machine learning algorithms in this paper. The collab runs the code on local hardware. Thus, execution time may vary from system to system.

### 3.2. Data Acquisition

**Figure 5:**
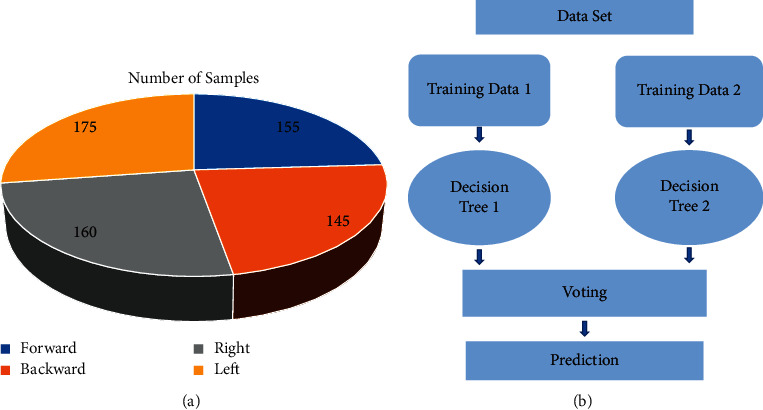
(a). Ratio of samples for postures, including forward, backward, left, and right. (b) Flow diagram 1: random forest working.

The data is collected at COMSATS UNIVERSITY Islamabad, Lahore campus, and students of different weights, heights, and body mass contributed to this study. The teachers and staff also contributed to dataset generation. Hence, the age ranges from 20 to 45 years. The number of samples collected is 635 different persons, and among them, 155 samples are forward-leaning, 145 are backward-leaning, 160 are right-leaning, and 175 are left-leaning, as shown in Figure 5(a). The BMI range of collected sample data varies from 16 to 35.

### 3.3. Data Preprocessing

Data preprocessing is an efficient technique to make raw data valuable and significant. Data preprocessing techniques refine the dataset into a functional form in this study. The repeated, corrupted, and null entries are eliminated from the dataset. Decreasing the irrelevant column is a significant step in removing the data and time columns from the dataset. Moreover, outliers that lead to the wrong prediction are removed. Another major step in data preprocessing is extracting null rows and columns and then removing them and placing them by their primary values. The smote analysis also removes the values where repetition exists to make the dataset more significant.

### 3.4. Data Processing

After the preprocessing of data, four machine learning algorithms are applied to detect the posture, and their accuracy and execution time are compared. Logistic regression predicts the output, which is present in categorical form. It can be either Yes or No, 0 or 1, true or False, etc., and it gives a value between 0 and 1. When applying logistic regression, the output is in categorical form. Logistic regression gives a value between 0 and 1, and it cannot go above this limit, forming an S-like curve. The S-form curve is called the Sigmoid function or the logistic function [32]. The probability of a record belonging to the positive class given feature prediction by the logistic regression (9).

(1) $$\begin{array}{c} P=\frac{1}{1+e^{-\left(\beta_{0}+\beta_{1}X_{1}+\beta_{2}X_{2}+\beta_{3}X_{3}\ldots \ldots \ldots +\beta_{n}X_{n}\right)}}. \end{array}$$

The other algorithm used in this study is KNN. Using KNN, the quick identification of the category or class is achieved [33]. The working of the KNN algorithm is as follows:

- Selecting the number *K* of the neighbors
- Euclidean distance of *K* number of neighbors is calculated
- The *K* nearest neighbors from the calculated Euclidean distance
- Counting the number of data points from *k* neighbors in each category
- For which number of the neighbor is maximally assigning them a new data point

Distance functions used to calculate the distance from the nearest neighbor are Euclidean (2), Manhattan (3), and Minkowski (4).

(2) $$\begin{array}{c} \text{Euclidean}=\sqrt{\overset{k}{\underset{i=1}{\sum }}{\left(x_{i}-y_{i}\right)}^{2}}, \end{array}$$

(3) $$\begin{array}{c} \text{Manhattan}=\overset{k}{\underset{i=1}{\sum }}\left|x_{i}-y_{i}\right|, \end{array}$$

(4) $$\begin{array}{c} \text{Minkoswski}={\left({\overset{k}{\underset{i=1}{\sum }}\left(\left|x_{i}-y_{i}\right|\right)}^{q}\right)}^{1/q}. \end{array}$$

The working diagram of the random forest is as shown in a flow diagram in Figure 5(b).

Another algorithm used in this study is Naïve Bayes. In the naïve Bayes algorithm, the occurrence of a certain feature is independent of the occurrence of other features [34].

The working of the naïve Bayes algorithm is as follows:

1. Converting the given dataset into frequency tables
2. Likelihood table generation by finding the probabilities of given features
3. For calculating the posterior probability using the Bayes theorem

**Figure 6:**
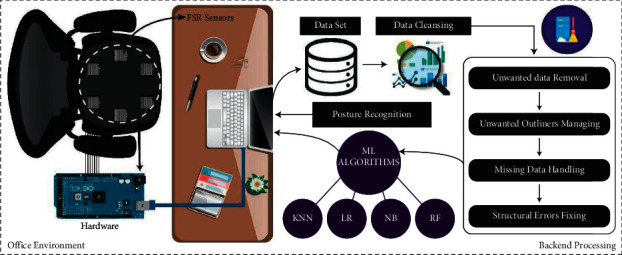
Complete system overview, including working and back-end environment.

The highest accuracy provider algorithm is random forest. Both big and small data can be handled using random forest. The random forest is a combination of multiple decision tree algorithms [35].  Figure 6 illustrates a complete overview, including working and back-end environment of the proposed system.

KNN is a lazy learner that stores data at the time of classification, however, the advantage of using KNN is that it is straightforward to implement. The logistic regression classification algorithm gives about 86.78% precision and an accuracy of 83.34%, just in a training time of 0.029 s. Logistic regression produces accurate results using binary classification tasks, while the proposed system consists of multiclass classification. Random forest contains several decision trees consisting of various subsets in the given dataset. It takes an average to improve the predictive accuracy of that dataset. As the number of trees increases in the forest, it leads to higher accuracy and prevents the problem of overfitting. Random forest generates a predicting precision of 97.3%, an accuracy of 97.08%, and the training time for data is just 0.662 s. Naïve Bayes generates the predicting precision of 82.22%, the accuracy of 77.38%, and the training time for data is just 1.5 s. It is a probabilistic classifier, which means it predicts based on the probability of an object. The examples of the Naïve Bayes algorithm are spam filtration, sentimental analysis, and classifying articles. Arrangement 01 results and comparison of different algorithms is given in Table 4.

## 4. Results and Discussion

Machine learning algorithms are used in this research to predict four improper sitting postures. Two different arrangements are used to study the accuracy and cost-efficient system. This designed system has evaluated classification algorithms, including logistic regression, Naive Bayes, KNN, and random forest. Classifiers used in this system are judged based on their performance parameters, including precision, accuracy, and training time.

### 4.1. Results of Arrangement 01

**Figure 7:**
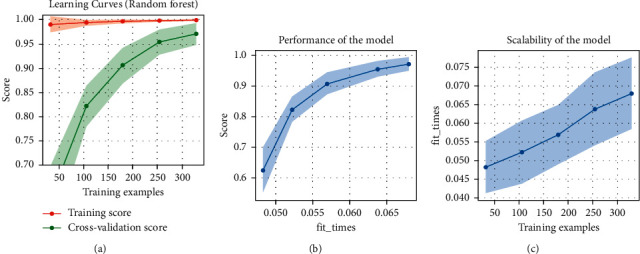
Random forest graphs: (a) learning curves, (b) performance of the mode, and (c) scalability of the model.

For this arrangement, the KNN (k-nearest neighbor) algorithm predicting precision is 89.8%, accuracy is 93.97%, and execution time is about 0.552 s. KNN is a nonparametric algorithm. It never makes any assumption on underlying data.  Figure 7 shows the plots of random forest algorithm for arrangement 01.

### 4.2. Arrangement 02 Results

**Figure 8:**
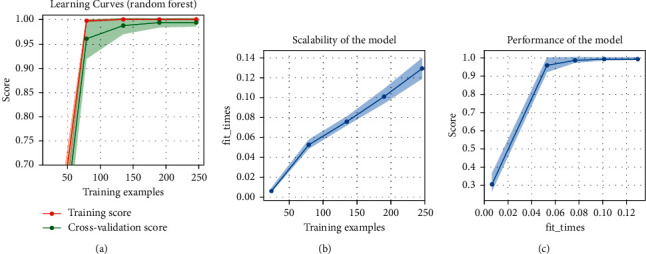
Table 3: arrangement 02: random forest graphs: (a) learning curves, (b) scalability of the model, and (c) performance of the model.

In this arrangement of sensors, two FSR and only one ultrasonic sensor are used. For sonar configuration, KNN (k-nearest neighbor) algorithm predicting precision is 99.97%, accuracy is 99.97%, and its execution time is about 0.519 s. The Logistic Regression classification algorithm gave a precision of about 99.96% and an accuracy of 99.97%, just in a training time of 0.029 s. Random forest generates the predicting precision of 99.78%, the accuracy of 99.998%, and the training time for data is just 0.062 s. Naïve Bayes generates the predicting precision of 82.22%, the accuracy of 77.38%, and the training time for data is just 1.5 s.  Figure 8 and Table 2 show the plots of random forest algorithm for arrangement 02 and comparison, respectively. (Table 5)

## 5. Conclusion and Future Work

In this era of technology, everything and every solution is just a click away on technical gadgets and computers. Nevertheless, this technology stuff somewhere has a significant impact on the overall health factors of the human body. This study presents a solution to the improper sitting habits of any person who works a lot sitting at a desk. In this study, FSR sensors and an ultrasonic sensor are used to detect the sitting posture of a human body. Four machine learning algorithms are applied to the dataset generated on four different improper sitting postures: forward-leaning, backward-leaning, left-leaning, and right-leaning. The accuracy of 99.998% is achieved using the Random forest machine learning algorithm when applied to arrangement 02 data set: two FSR sensors and one ultrasonic sensor. The system can be used in offices and at home during desk work to prevent improper posture hazards and maintain fitness. This paper's vital scientific contributions are as follows: (1) compared to flex and textile sensor systems, the proposed technique reduces the number of sensors and processing complexity, resulting in lower hardware overhead. This energy-efficient and trustworthy continuous sitting position detection system will play a more critical role in reducing musculoskeletal disorders and protecting users' dignity. (2) The proposed solution does not have a drift problem, and hence, repetitive calibrations may be avoided. As a result, it is more dependable and power-efficient than the accelerometer-based system, resulting in a longer battery life. (3) The proposed approach provides a more private user experience than the camera-based system. For future work, the communication can be done wirelessly to send the sitting posture history to health professionals and analyze in a better/faster way to understand the medical history. Furthermore, the real-time posture detection on a smartphone can be done as it is easy to access and efficient to detect the posture while a person sits anywhere rather than specifically on an office chair or work desk.

## Figures and Tables

**Table 1:** Literature review.

Sr no	Paper	Type of sensors	No. of postures	Classifiers/software	Accuracy (%)
1	[3]	A film-type 8 × 8 FSR	5	(CNN), (DT), (SVM), (MLR), (NN), and (NB)	95.3
2	[11]	Inverse piezoresistive nanocomposite sensors	3	Three-layer BP neural network	98.75
3	[9]	Pressure sensor of film type	7	(CNN), (ANN)	97.5
4	[10]	An array of six flex sensors	7	Artificial neural network	97.8
5	[12]	16 sensors with 16 matrices	4	k-nearest neighbors (k-NN), support vector machines (SVM), random forest (RF), decision tree (DT) and LightGBM	99.03
6	[31]	Accelerometer, gyroscope, and magnetometer sensors	5	Naive Bayes, SVM, and KNN	99.90

**Table 2:** Hardware component details.

Hardware	Specification
Arduino mega	Arduino mega is a microcontroller kit for building digital devices based on the ATmega 2560. It has 54 digital input/output pins
Laptop	A laptop used for data collection and processing has the following specifications: intel core i5, 6^th^ generation, G3 2.40 GHz processor, and 8 GB ram
Force-sensitive resistance	A force-sensitive resistor (FSR) is a material that changes its resistance, when a force or pressure is applied, the output voltage varies from 0 to 5 V, depending on the amount of force applied to the sensor
Sonar sensor	Sonar sensors use ultrasonic sound waves to detect objects and distance. It sends an ultrasonic pulse out at 40 kHz, which travels through the air, and if there is an obstacle or object, it will bounce back to the sensor

**Table 3:** Cost comparison of arrangement 01 and arrangement 02 of sensors distribution.

Components		Arduino UNO	Arduino mega	FSR Sensors	Ultrasonic Sensors	Total
Arrangement 01	Quantity	0	1	6	0	73.08$
	Cost	0	35.88$	6.2 × 6 = 37.2$	0	

Arrangement 02	Quantity	1	0	2	1	37.89$
	Cost	24.95$	0	6.2 × 2 = 12.4$	0.54$	

**Table 4:** Arrangement 01, machine learning algorithms comparison.

Algorithms	Precision (%)	Accuracy (%)	Training time (s)
Logistic regression	86.78	83.34	0.029
Naïve bayes	82.22	77.38	1.5
KNN	89.8	93.97	0.552
Random forest	97.3	97.08	0.662

**Table 5:** Comparison of machine learning algorithms of arrangement 02.

Algorithms	Precision (%)	Accuracy (%)	Training time (s)
Logistic regression	99.96	99.98	0.029
Naive bayes	82.22	77.38	2.56
KNN	99.97	99.97	0.519
Random forest	99.78	99.998	0.062

## Data Availability

No data were used to support this study.
